# Temporary abstinence challenges: What do we need to know?

**DOI:** 10.1111/dar.13625

**Published:** 2023-02-19

**Authors:** Anna Butters, Inge Kersbergen, John Holmes, Matt Field

**Affiliations:** ^1^ Department of Psychology University of Sheffield Sheffield UK; ^2^ School of Health and Related Research University of Sheffield Sheffield UK

## Abstract

Participation in temporary abstinence challenges (TAC) continues to increase with campaigns established in several countries. Temporarily abstaining from alcohol as part of such challenges is associated with ongoing benefits including reductions to alcohol consumption after the TAC. We identified three research priorities regarding TACs which are outlined in this paper. First, the role of temporary abstinence itself is unclear with post‐TAC reductions in alcohol consumption still apparent among participants who do not remain fully abstinent throughout the challenge. It is necessary to establish to what degree temporary abstinence itself, rather than the combination of abstinence and the additional supports provided by TAC organisers (e.g., mobile applications, online support groups), contributes to changes in consumption after the TAC. Second, little is known about the psychological changes underlying these changes in alcohol consumption, with conflicting evidence as to whether increases in someone's belief in their ability to avoid drinking mediates the association between participation in a TAC and reductions in consumption afterwards. Other potential psychological and social mechanisms of change have been subjected to little, if any, scrutiny. Third, evidence of increased consumption post‐TAC among a minority of participants indicates a need to establish for whom or in what circumstances participation in a TAC may result in unintended negative consequences. Focussing research in these areas would increase the confidence with which participation could be encouraged. It would also enable campaign messaging and additional supports to be prioritised and tailored to be as effective as possible in facilitating long‐term change.

## 1 INTRODUCTION

Temporary alcohol abstinence challenges (TAC) have increased in popularity over the past decade with campaigns established in Europe, North America, Thailand and Australia [1, 2, 3, 4, 5, 6, 7, 8, 9]. Large numbers of people sign up to these campaigns, with many more participating informally without accessing the additional supports provided by organisers [1, 10]. For example, in 2021, 130,000 UK drinkers registered for the official ‘Dry January’ campaign, with an estimated 6.5 million also taking part informally without registering [1]. Participation has been to linked to health benefits which may persist beyond the period of the TAC itself, with some participants reducing their alcohol consumption when they resume drinking after the TAC has ended [10, 11, 12, 13, 14].

Given their extensive reach, popularity, simplicity and potential for long‐term benefits, TACs may provide a low‐cost way to reduce alcohol harms at the population‐level. It is therefore important to establish *whether*, *how*, *for whom* and *in what contexts* participation in TACs leads to long‐term behaviour change. This would enable organisers to tailor campaign messaging and maximise the effectiveness of the supports provided. In this paper we outline the need for well‐controlled research to confirm the outcomes of participation, investigate the mechanisms through which they prompt people to change their drinking, and evaluate their potential for unintended adverse effects.

## 2 OUTCOMES OF PARTICIPATION

Evaluations of 1‐month TACs found that participation was associated with an enduring reduction in alcohol consumption [10, 11, 12, 13, 14, 15]. Prospective studies of Dry January in the United Kingdom [11, 14], IkPas in the Netherlands [10, 12] and Tournée Minérale in Belgium [13] found participation to be associated with a reduction in alcohol consumption 6 months after the challenges were over, with variable effect sizes reported (e.g., *d* > 0.18 in [14]). Studies of Dry January also identified increases in drink refusal self‐efficacy (DRSE; i.e., confidence in the ability to refuse alcohol [16]) at 1‐month follow‐up [11, 14], alongside improvements in wellbeing, and mental and physical health [11, 17, 18, 19].

Outside of organised TACS, temporary abstinence has been associated with improvements in physical health markers [20, 21]. However, these improvements were not maintained following the resumption of drinking [20], indicating the importance of studying ongoing changes to drinking behaviour post‐TAC. Two prospective studies compared TAC participants with drinkers who were not attempting to temporarily abstain. Although drinkers not participating in a TAC did not reduce their drinking over time [11, 13], TAC participants reported significantly higher alcohol consumption at baseline compared to the control group. Therefore, any reduction in consumption at follow‐up in TAC participants could be partially attributable to regression to the mean [22].

## 3 DISENTANGLING THE ROLES OF TEMPORARY ABSTINENCE, COMMITMENT AND EXTERNAL SUPPORTS

Prospective studies revealed that TAC participation is associated with reduced alcohol consumption at follow‐up and improved mental health and wellbeing, even in participants who did not completely abstain (although effects were larger in TAC participants who did so) [11, 14, 18, 19]. This suggests that while abstinence is the focal point of campaigns, it is not the only factor contributing to longer‐term outcomes. This is unsurprising as many TACs are complex interventions with multiple components which complement as well as facilitate abstinence. These components, including registration, mobile phone applications for goal‐setting and progress monitoring, and online peer support groups, may be active ingredients that individually or in combination promote long‐term behaviour change.

For example, participants are more likely to remain abstinent throughout a TAC if they commit to doing so by formally registering [11, 23, 24, 25]. These findings are consistent with the broader literature on commitment and health behaviour change [26, 27, 28]. People who registered for Dry January but did not remain abstinent were more likely to have improved DRSE and wellbeing scores compared to informal participants who did remain abstinent [29]. This highlights the relative roles of commitment and temporary abstinence and the possibility that registering for a TAC may contribute to long‐term change, even if abstinence is not maintained. Future studies should evaluate the association between duration and/or frequency of periods of abstinence and changes to alcohol consumption at follow‐up. This would establish if there are particular patterns of abstinence that are associated with long‐term benefits; in turn this may enable briefer TACs (e.g. 1 week) to be introduced, which might increase their acceptability.

The picture is further complicated by the external supports that are sometimes offered to registered participants (e.g., [1, 4, 7, 8]). Engagement with this support may be important: participants who read daily support emails were more likely to remain abstinent during Dry January than those who did not [18]. Having access to such supports may also help participants manage challenges associated with temporary abstinence including lack of support, social consequences and the inescapability of alcohol in society [30]. While studies have examined the frequency of use of various supports [12, 13, 17, 29] and begun to explore their role in supporting TAC participants through the abstinence period [17, 31, 32] it remains unclear how *use of*, rather than *access to*, supports is associated with enduring changes to alcohol consumption.

Temporary abstinence, commitment and external supports may all play a role in the outcomes associated with TAC participation. Disentangling the contributions of these different factors could influence how TACs are framed to prospective participants. For example, if successfully completing the period of abstinence is key to long‐term benefits, then a simple message—‘try taking a month off alcohol’ could be incorporated into public health messaging, brief interventions, health service websites and other population‐level campaigns. Alternatively, if commitment or use of supports is more important than temporary abstinence, campaign messaging could encourage people to register and actively engage with the supports available.

## 4 MECHANISMS OF CHANGE

We have limited understanding of the psychological and social mechanisms underpinning any longer‐term reductions in alcohol consumption after TACs. One candidate mechanism is DRSE, with increased DRSE following participation in Dry January associated with reduced alcohol consumption [14], consistent with the broader literature [33, 34, 35]. However, this finding has not been consistently replicated across other TACs [13] and should be further scrutinised.

Other potential determinants of change following TAC participation have yet to be examined. Motivation to change is an important modifiable determinant of behaviour, although its role in alcohol‐related behaviour change is complex and ambiguous [36, 37, 38]. TAC participants are a self‐selected group, many of whom are likely motivated to reduce their alcohol consumption. Indeed, Dry January participants are more concerned about their drinking compared to drinkers who do not participate [11]. Furthermore, the majority of TAC participants want to make changes to their drinking beyond the TAC itself [10, 12, 17]. However, not everyone who participates in a TAC does so with the intention of making long‐term changes to their drinking. While some TACs are promoted as a way to break habits and change one's relationship with alcohol (e.g., [1, 4, 5, 7, 8]) others are predominantly framed as fundraising challenges (e.g., [2, 3]). The framing of a TAC may influence who participates and whether they aspire to make longer‐term changes to their drinking [15, 29]. For example, around 40% of febfast 2011 (fundraising focussed) participants reported taking part to initiate an ongoing change [15], compared to 97% of registrants surveyed prior to Dry January 2019 (behaviour change focussed) [29]. Therefore, the framing of campaigns might contribute to the probability of participants making ongoing changes to their alcohol intake, suggesting a need for comparative analyses across TACs or between distinct iterations of a single TAC. Overall, the role of motivation to change, both as a determinant of who signs up for TACs and as a potentially enduring consequence of participation, requires further study.

Other notable determinants of health behaviour change include identity change, modification of social routines and increasing recognition of the health consequences of a behaviour [39, 40]. As applied to TACs, changes to drinker identity [31], adjustment of social practices to accommodate ongoing changes to alcohol consumption [41] and experiencing the health benefits of temporary abstinence [11, 29, 42] may all contribute to enduring reductions in drinking following TAC participation; all are worthy of further investigation. Understanding the mechanisms through which participation in TACs leads to change would enable organisers to prioritise, develop and refine intervention components to target the relevant determinants and thereby increase the likelihood of participants reducing their drinking over the longer‐term.

## 5 POTENTIAL NEGATIVE EFFECTS

It is important to look beyond the intended outcomes of a behaviour change intervention and consider the potential for it to have unintended negative consequences such as rebound effects and risk compensation [43, 44]. People with alcohol dependence are discouraged from participating in TACs because of the risks associated with abrupt abstinence without medical supervision. Separately, concerns have been raised that some risky drinkers may engage in temporary abstinence to justify hazardous drinking throughout the rest of the year [45]. This has received little research attention although one previous study indicates it may only apply to a small minority of TAC participants, who report an increase in frequency of drunkenness 6‐months later [14]. Nonetheless, it is essential to establish whether, for some people, participating in a TAC could discourage them from reducing their drinking longer‐term, and whether the way in which TACs are framed, for example, as a standalone fundraising challenge rather than a way to initiate ongoing change, contributes to this. Characterisation of individual differences that predict negative outcomes during and after participation could enable such individuals to be identified, discouraged from TAC participation and directed to alternative support.

## 6 SUMMARY

TACs have become increasingly popular. They offer potential for a relatively low‐cost way of tackling alcohol‐related harms, but to fulfil this potential it is crucial that we develop a deeper understanding of them. The popularity and longevity of TACs ensures a living laboratory through which we could further our understanding of these complex interventions using sophisticated prospective observational and randomised studies to confirm the apparent benefits of taking part and to clarify the mechanisms through which participation leads to ongoing changes to drinking. We must also attempt to determine the likelihood of unintended negative consequences in order to mitigate them. Establishing the extent to which TACs help people to reduce their drinking, for whom, and how, would ensure that messaging and the external supports provided can be tailored to maximise the potential benefits of participation while minimising any harms.

## AUTHOR CONTRIBUTIONS

Each author certifies that their contribution to this work meets the standards of the International Committee of Medical Journal Editors.

## CONFLICT OF INTEREST STATEMENT

Anna Butters's PhD studentship is funded by the Economic and Social Research Council and Alcohol Change UK who are the organisers of Dry January in the UK.
